# Supplementary material for: Establishment of Etv5 gene knockout mice as a recipient model for spermatogonial stem cell transplantation
Source: Biol Open. 2021 Jan 6;10(1):bio056804. doi: 10.1242/bio.056804 (PMC7803462; doi:10.1242/bio.056804)
Supplement: Supplementary information [file biolopen-10-056804-s1.pdf]

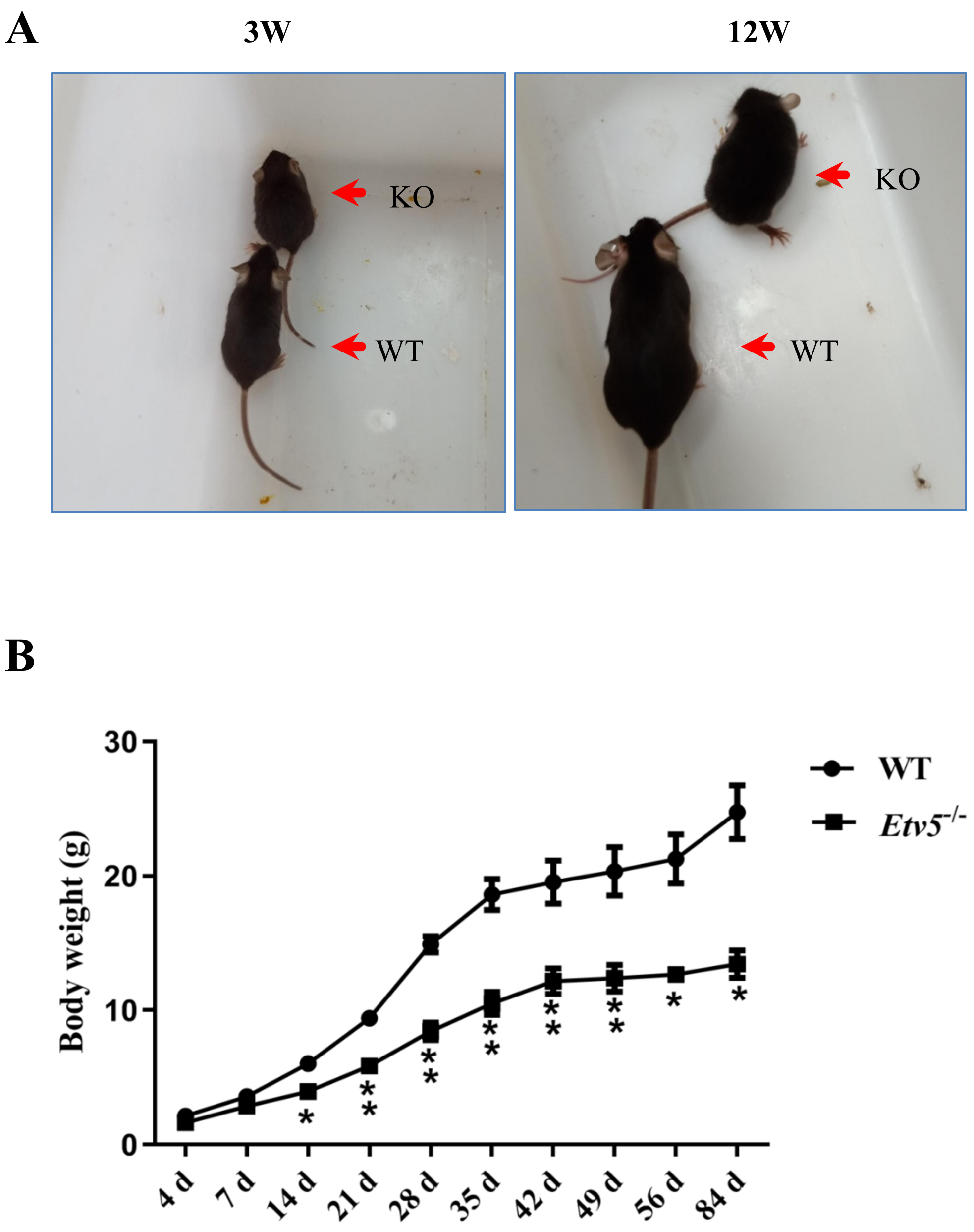

Supplementary Fig. 1

Fig. S1. Body weight of KO mice. (A) The phenotype of WT and *Etv5*$^{-/-}$ mice at 3 and 12 weeks of age. Arrows indicate *Etv5*$^{-/-}$ and WT mice. (B) Body weight of WT and *Etv5*$^{-/-}$ mice at periodic time points during development from 4 postnatal days to adulthood (12 weeks). For all time points, n = 5 for both WT and *Etv5*$^{-/-}$ mice. Data presented as mean ± S.D. *$P < 0.05$, **$P < 0.01$, assessed by unpaired *t*-test.

Biology Open • Supplementary information

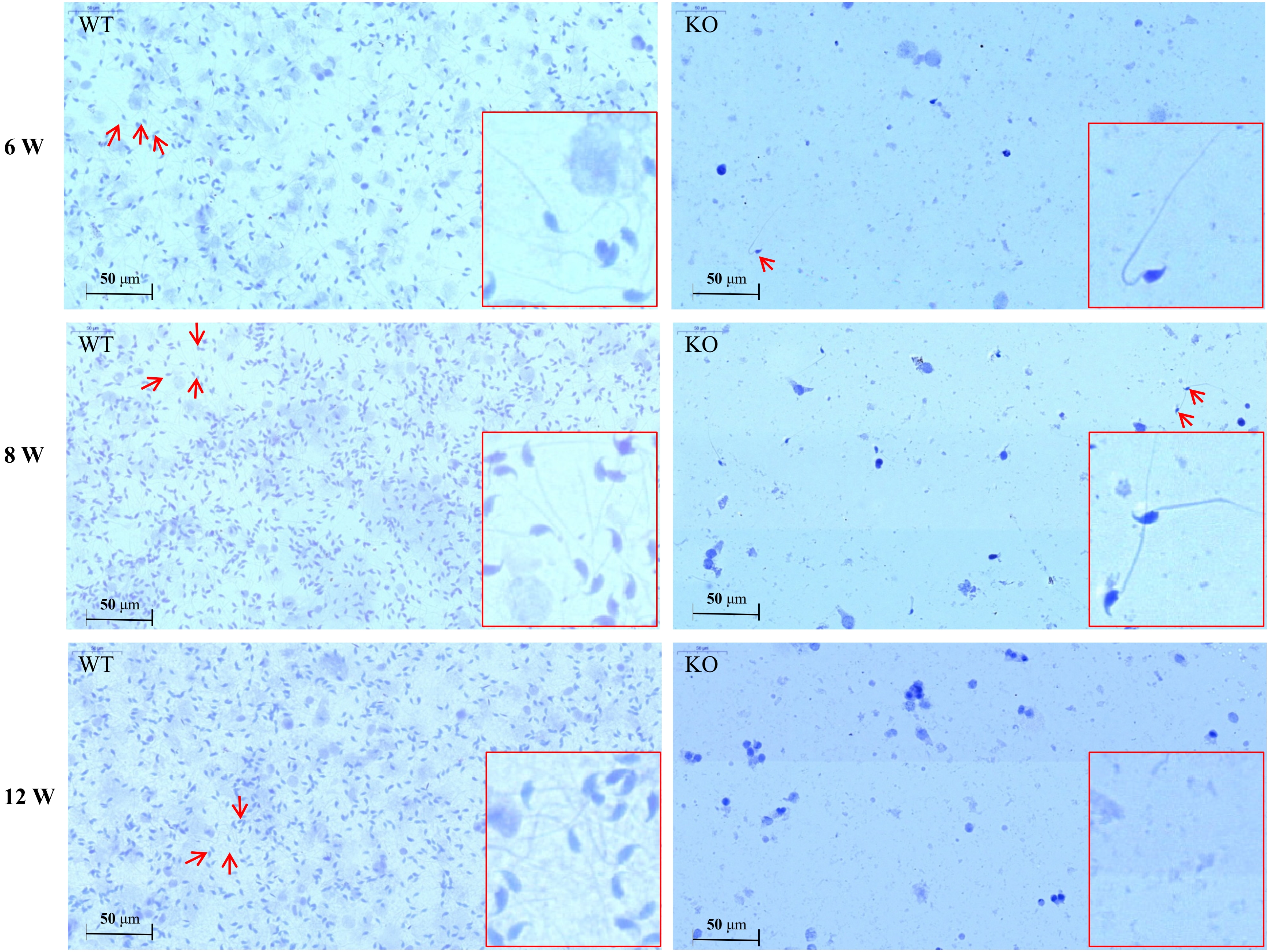

Supplementary Fig. 2

Biology Open • Supplementary information

Fig. S2. Sperm density in the epididymis in 6-, 8-, and 12-week WT and *Etv5*$^{-/-}$ mice. Arrows indicate sperm and the insert represents the higher magnification of sperm. Scale bars = 50 µm.

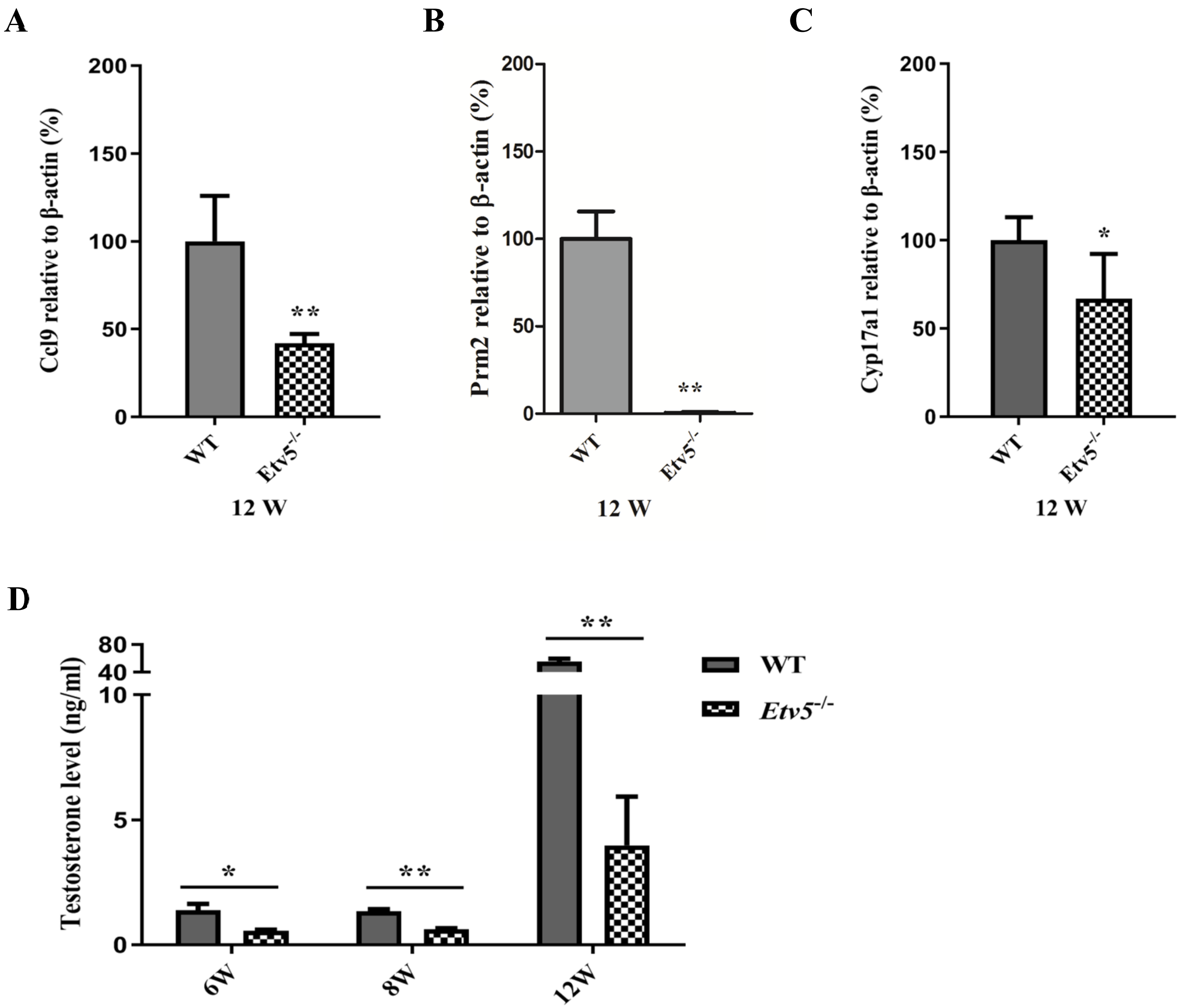

Supplementary Fig. 3

Biology Open • Supplementary information

Fig. S3. Changes in gene expression related to spermatogenesis and testosterone concentrations. mRNA expression levels of *Etv5* target gene *Ccl9* (A), spermatids-specific gene *Prm2* (B) and interstitial gland-specific gene *Cyp17a1* (C) in the testes of WT and *Etv5*$^{-/-}$ mice littermates at 12 weeks. WT values were set as 100%. (D) Testosterone concentrations of WT and *Etv5*$^{-/-}$ littermates at 6, 8 and 12 weeks. n = 3 per group. Data presented as mean ± S.D. **P*＜0.05, ***P*＜0.01, assessed by unpaired *t*-test.

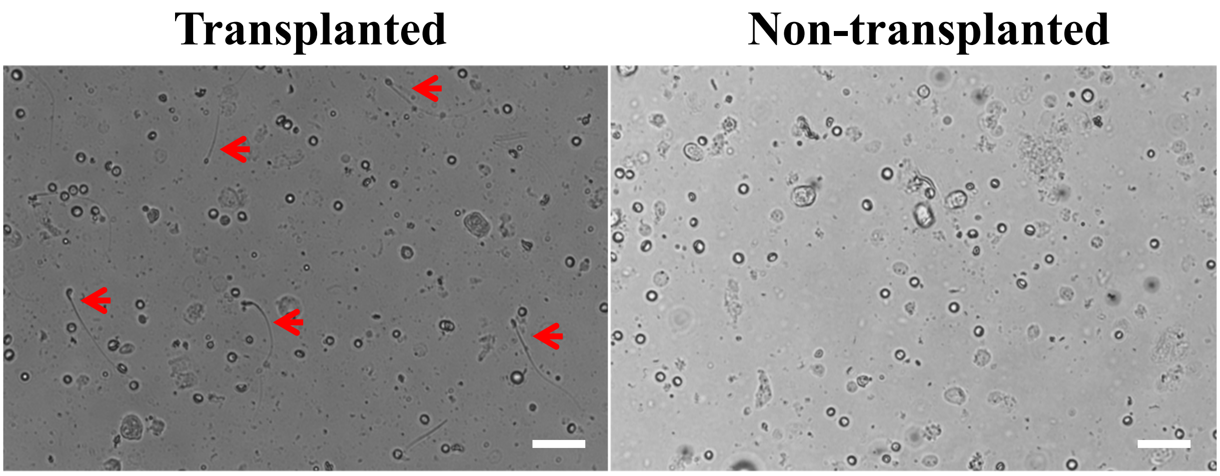

Supplementary Fig. 4

Fig. S4. Sperm regeneration. The sperm collected from epididymis in recipient mice 2 months post-transplantation. Mature sperm with intact shape can be observed (Red arrows). Scale bars = 50 µm.

Biology Open • Supplementary information
